# Biomimetic phantom for cardiac diffusion MRI

**DOI:** 10.1002/jmri.25014

**Published:** 2015-07-24

**Authors:** Irvin Teh, Feng‐Lei Zhou, Penny L. Hubbard Cristinacce, Geoffrey J.M. Parker, Jürgen E. Schneider

**Affiliations:** ^1^ Division of Cardiovascular MedicineRadcliffe Department of MedicineUniversity of OxfordOxfordUK; ^2^ Centre for Imaging Sciences, Manchester Academic Health Sciences Centre, University of ManchesterManchesterUK; ^3^ The School of Materials, University of ManchesterManchesterUK; ^4^ Biomedical Imaging InstituteUniversity of ManchesterManchesterUK

**Keywords:** cardiac MRI, diffusion tensor imaging, biomimetic phantom, co‐electrospinning

## Abstract

**Purpose:**

Diffusion magnetic resonance imaging (MRI) is increasingly used to characterize cardiac tissue microstructure, necessitating the use of physiologically relevant phantoms for methods development. Existing phantoms are generally simplistic and mostly simulate diffusion in the brain. Thus, there is a need for phantoms mimicking diffusion in cardiac tissue.

**Materials and Methods:**

A biomimetic phantom composed of hollow microfibers generated using co‐electrospinning was developed to mimic myocardial diffusion properties and fiber and sheet orientations. Diffusion tensor imaging was carried out at monthly intervals over 4 months at 9.4T. 3D fiber tracking was performed using the phantom and compared with fiber tracking in an ex vivo rat heart.

**Results:**

The mean apparent diffusion coefficient and fractional anisotropy of the phantom remained stable over the 4‐month period, with mean values of 7.53 ± 0.16 × 10^‐4^ mm^2^/s and 0.388 ± 0.007, respectively. Fiber tracking of the 1st and 3rd eigenvectors generated analogous results to the fiber and sheet‐normal direction respectively, found in the left ventricular myocardium.

**Conclusion:**

A biomimetic phantom simulating diffusion in the heart was designed and built. This could aid development and validation of novel diffusion MRI methods for investigating cardiac microstructure, decrease the number of animals and patients needed for methods development, and improve quality control in longitudinal and multicenter cardiac diffusion MRI studies. J. MAGN. RESON. IMAGING 2016;43:594–600.

Heart function is underpinned by the contraction of individual cardiomyocytes and their organization as a highly ordered functional syncytium. Characterization of this complex tissue microarchitecture as a basis for understanding cardiac function is both important and challenging.^1^ Diffusion magnetic resonance imaging (MRI) measures the diffusion of water,^2^ which is hindered and restricted in the cellular environment, and consequently serves as a marker of cellular structure and organization in biological tissues.^3^ Diffusion tensor imaging (DTI) is a rapidly emerging and noninvasive approach^4^ for assessing cardiac microstructure. Still, there remain many challenges in its implementation in vivo. These include limited signal‐to‐noise ratio (SNR), limited imaging resolution, long scan times, and sensitivity to motion, strain, and eddy currents.

The rapid growth of diffusion MRI, within and outside the field of cardiac imaging, has driven the demand for phantoms to aid development of novel pulse sequences and models of diffusion. However, it is difficult to exclude the effects of motion and strain in dynamic experiments. As such, investigations focusing on isolating the effects of parameters independent of motion may be better served by using static phantoms. In providing a stable substrate with biologically relevant, and known, diffusion properties, phantoms can help assess the performance of novel methods free of motion and at high SNR. Independent measurement of phantom structure using high‐resolution microscopy can be used to establish ground truth data and increase confidence in methods and hardware used to acquire and analyze diffusion MRI data.

The majority of diffusion phantoms^5^, ^6^, ^7^, ^8^, ^9^, ^10^, ^11^ have been 1) designed to simulate diffusion in the brain, and 2) utilize solid fibers, which contrasts with the cellular structure of tissue containing a distribution of pores. One promising method based on co‐electrospinning produces hollow microfibers, which are perfused with an MRI‐visible substrate.^12^ This yields physiological values of mean apparent diffusion coefficient (ADC) and fractional anisotropy (FA), which can be customized based on the specified pore size.^13^ These phantoms have previously been developed as mimics of diffusion in neurological white matter. In this study we designed and fabricated a static phantom mimicking diffusion in the myocardium, so as to aid development of cardiac diffusion MRI methods and improve quality control in longitudinal and multicenter studies.

## Materials and Methods

### Phantom Design and Fabrication

**Figure 1:**
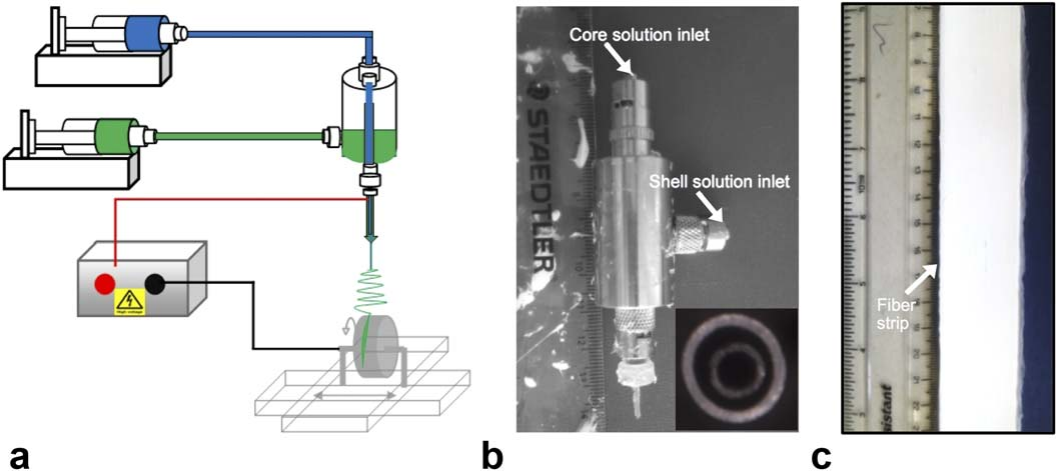
**(a)** Schematic of setup for co‐electrospinning of microfiber strips, **(b)** coaxial spinneret with separate inlets for the core and shell solutions (inset: spinneret tip), and **(c)** photo of the fiber strip. With kind permission from Springer Science and Business Media, Macagnano A, et al. (eds), Electrospinning for high performance sensors. 2015.

Co‐electrospinning was performed using a vertical setup comprising two syringe pumps, two syringes, a concentric spinneret, a high‐voltage power source, and a grounded drum mounted on an x‐y translation stage serving as a fiber collector (Fig. 1). The two plastic syringes were mounted on pumps and filled with polycaprolactone (PCL) and polyethylene oxide (PEO) solutions, with independently controlled PEO and PCL flow rates. The syringes were connected by plastic tubes and Luer‐Lock connections to the inlets of the coaxial spinneret. PEO solution flowed through the inner needle (ie, core fluid) and PCL solution flowed through the annular gap between the inner and outer needle (ie, shell fluid). As the electric voltage of the spinneret increased with respect to the grounded collector, two menisci formed at the tip of the spinneret in a conical‐like shape, with the shell meniscus surrounding the core one. Within a suitable range of core and shell fluid flow rates and applied voltage, these menisci would give way to an electrified coaxial jet of polymer. After stretching and evaporation of core and shell solvents, the polymer was progressively deposited on the simultaneously rotating and translating drum collector to form a continuous band of hollow microfibers measuring 30 mm wide and 0.5 mm thick. More detail on the optimization of the co‐electrospinning method can be found elsewhere.^12^, ^13^, ^14^

**Figure 2:**
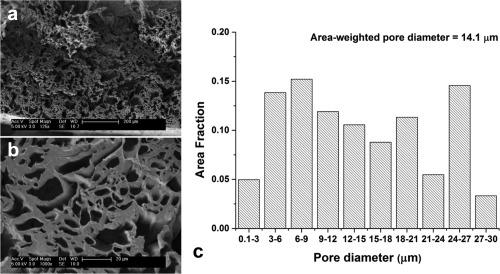
Scanning electron micrographs depict a cross‐section of the fiber strip at **(a)** 125× and **(b)** 1000× magnification. **(c)** A histogram illustrates the measured pore size distribution across 10 SEM images (1000× magnification) in the fiber strip.

A 5‐cm long section was cut for scanning electron microscopy (SEM) characterization. Cross‐sections of fiber strips were prepared using a scalpel in liquid nitrogen and coated with thin gold film to increase their conductivity for SEM. They were examined with a Philips (Best, Netherlands) XL30 FEG SEM with an accelerating voltage of 5 kV. Fiber inner diameters were measured in 10 SEM images at 1000× magnification using the Binary and Analyze Particles functions of ImageJ (NIH, Bethesda, MD). The area‐weighted average fiber diameter and fractional area histograms of fiber diameters were measured (Fig. 2).

**Figure 3:**
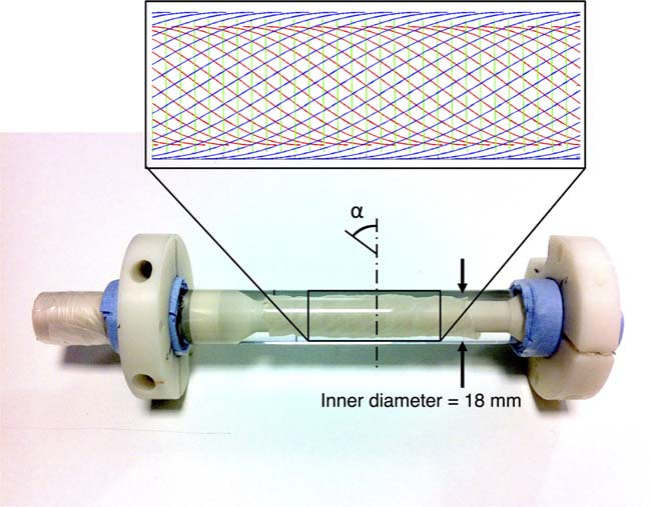
Photograph of cardiac diffusion phantom comprising three layers of fiber strips wound at different helix angles (α) concentrically about a central polymer spindle. The larger diameter supports at the ends of the spindle fit closely in the glass tube for improved stability, and a bubble trap was incorporated near the mouth of the tube. The inset shows a schematic diagram of the primary fiber orientation in the inner (red), middle (green), and outer (blue) fiber strips. Their effective radii of curvature were 273, 4.88, and 526 mm, based on the measured mean helix angles of 52°, 2.0°, and –58°, respectively.

The remaining length of fiber strip was cut into three sections and perfused with cyclohexane, the MRI‐visible diffusate, via immersion in cyclohexane for 1 hour at room temperature. These were wound concentrically about a custom polyphenylsulphide spindle at three different angles to simulate the right, circumferential, and left helical fibers of the myocardium in the left ventricle (LV), progressing from the subendocardium to the subepicardium (Fig. 3). Each layer was held in place by a 20 μm diameter unifilament winding of Dyneema (DSM, Netherlands). The spindle and fibers were then inserted horizontally into an NMR tube of 18 mm inner diameter containing cyclohexane and sealed off with two Viton O‐rings. All preparation was performed while immersed in a bath of cyclohexane to exclude air bubbles. Any residual bubbles were contained in the bubble trap built into the spindle.

### Data Acquisition

MRI was performed using a 9.4T preclinical scanner (Agilent Technologies, Santa Clara, CA), a shielded gradient system, and a transmit/receive birdcage coil of 20 mm inner diameter (Rapid Biomedical, Rimpar, Germany). 2D spin echo (SE) DTI was performed at monthly intervals over 4 months to assess sample stability with the following sequence parameters: relaxation time / echo time (TR/TE) = 2500/17.5 msec, in‐plane resolution = 0.15 × 0.15 mm, slice thickness = 1 mm, number of slices = 17, δ = 2.5 msec, Δ = 12.5 msec, b_max_ = 2000 s/mm^2^, number of non‐DW images = 4, number of DW directions = 30. In addition, 3D fast spin echo (FSE) DTI data were acquired at the final timepoint for fiber tracking, with: TR/TE = 800/17.1 msec, resolution = 0.2 × 0.2 mm, δ = 2.5 msec, Δ = 12.5 msec, b_max_ = 1000 s/mm^2^, number of non‐DW images = 2, number of DW directions = 15. To ensure consistency in slice positioning, the sample was kept in the coil over the 4‐month period. For comparison, ex vivo 3D FSE data were acquired in one excised rat heart. Experimental investigations conformed to the UK Home Office guidance on the Operations of Animals (Scientific Procedures) Act 1986 and were approved by the University of Oxford's Ethical Review Board.

### Data Analysis

A single tensor model was fit to the SE data using a linear least‐squares approach, and the mean ADC, FA, and helix angles across the simulated myocardium were measured using custom‐written code (MatLab R2013a, MathWorks, Natick, MA). A multislice mask comprising the phantom fibers was applied by thresholding each dataset at each timepoint at a fixed percentage of the respective global maximum b = 0 image signal intensity. Boxplots of mean ADC and FA in the masked region are displayed across a 4‐month period, and average, median, interquartile ranges, and coefficients of variation (CV) were calculated using MatLab R2013a. Fiber tracking was performed on the 3D FSE data using Diffusion Toolkit and Trackvis.^15^

## Results

**Figure 4:**
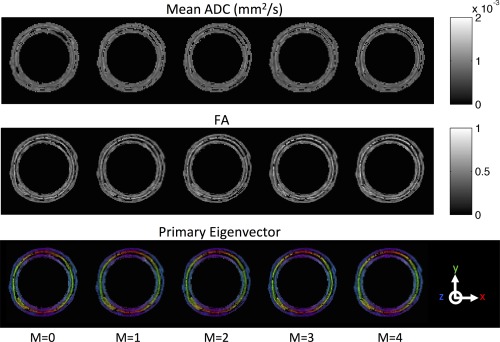
Mean ADC, FA, and primary eigenvector maps, in a mid‐axial slice at monthly intervals ranging from M = 0 to M = 4 (left to right). Masks based on a fixed percentage threshold of the global maximum signal intensity in the b = 0 images were applied. Primary eigenvectors oriented in the scanner x‐, y‐, and z‐axes are represented in red, green, and blue, respectively.

**Figure 5:**
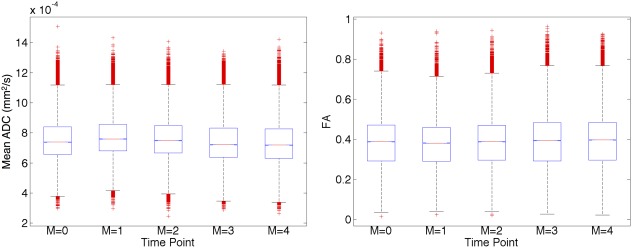
Boxplots of mean ADC and FA illustrate their stability over a 4‐month period. The values cited are based on data from 17 slices in the phantom, with masks applied based on a fixed percentage threshold of the global maximum signal intensity in the b = 0 images.

Mean ADC, FA, and primary eigenvector maps are shown in the middle slice of the SE data at five timepoints (Fig. 4). Boxplots of the mean ADC and FA, in the masked region across 17 slices, at each timepoint are presented (Fig. 5). These values remained stable over the 4‐month period. The average, median, interquartile range, and CV of the mean ADC were 7.53 ± 0.16 × 10^‐4^ mm^2^/s, 7.37 ± 0.17 × 10^‐4^ mm^2^/s, 1.87 ± 0.08 × 10^‐4^ mm^2^/s, and 2.07%, respectively, while the corresponding measures of the FA were 0.388 ± 0.007, 0.389 ± 0.006, 0.181 ± 0.009, and 1.67%, respectively. Data (mean ± standard deviation) are reported across the monthly intervals (*n* = 5). While the mean ADC and FA were relatively uniform in the inner and outer fiber layers, the mean ADC and FA in the middle layer were 11% lower and 18% higher, respectively. The middle layer was also thinner and exhibited lower signal intensity in the b = 0 images, relative to the inner and outer strips.

**Figure 6:**
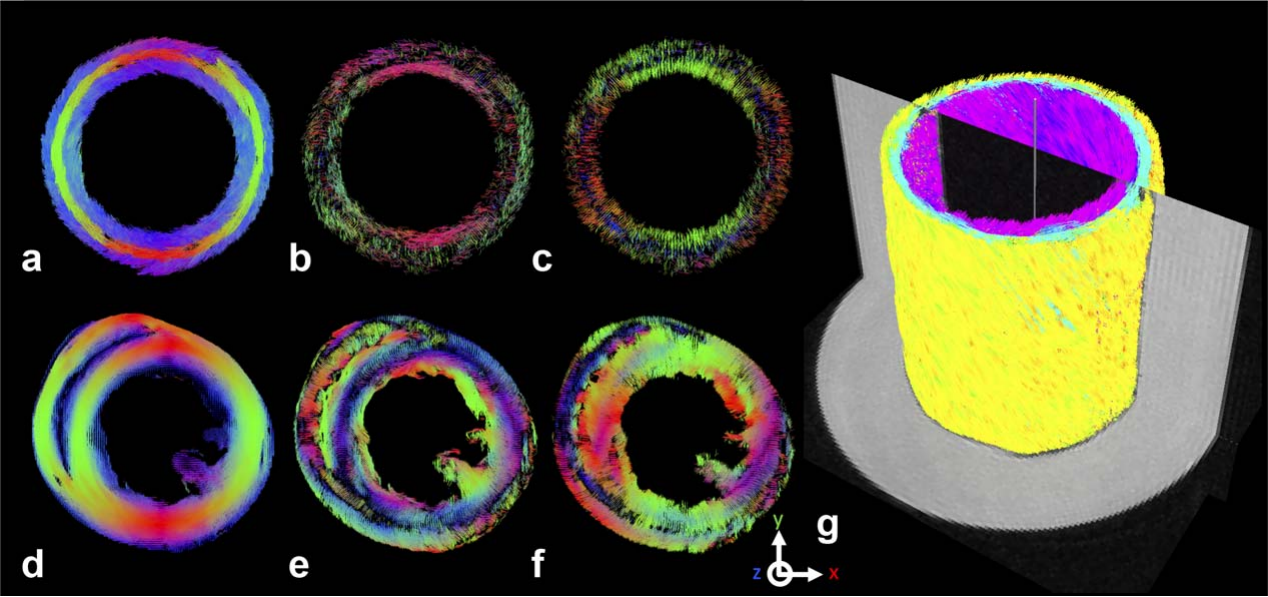
3D fiber tractography in a simulated mid‐myocardial short‐axis slice in the phantom based on the **(a)** primary, **(b)** secondary and **(c)** tertiary eigenvectors. **(d–f)** Corresponding data in an ex vivo rat heart are shown. Tracts oriented in the scanner x‐, y‐, and z‐axes are represented in red, green, and blue, respectively. The transition in primary fiber orientation across the (d) left ventricular myocardial wall thickness is replicated, in a stepwise manner, in the (a) phantom. The radial orientation of the tertiary eigenvectors in the (f) left ventricle, indicative of the sheet‐normal direction of the myocardial laminae, are likewise apparent in the inner and outer layers of the (c) phantom data. **(g)** Fiber tracts based on the primary eigenvector are shown in an isometric view, colorized by helix angle and superimposed on the b = 0 image. The tracts illustrate the transition from left helical fibers (negative helix angle; yellow) of the simulated subepicardium, through circumferential fibers (neutral helix angle; cyan) of the simulated mid‐myocardium, to right helical fibers (positive helix angle; magenta) of the simulated subendocardium.

Fiber tracking based on the 3D FSE data after 4 months illustrates how the phantom simulates the fiber and sheet orientation of the LV (Fig. 6). Tracts in the phantom, based on the 1st eigenvector, can be seen to transition from a right helical, through circumferential, to left helical arrangement simulating the transition observed in the subendocardium to subepicardium of the LV of the rat heart. The tracts in the phantom were color‐coded in 1) red, green, and blue representing the scanner coordinate system, and 2) cyan, magenta, and yellow to highlight the transition in helix angle. Mean helix angles of the simulated subendocardium, mid‐myocardium, and subepicardium were 52° ± 9°, 2° ± 8°, and –58° ± 9°, respectively. Fiber tracking was also performed based on the 2nd and 3rd eigenvectors. We observed that the 2nd eigenvector tracts in the inner and outer fiber strip were oriented in a reciprocal left helical and right helical arrangement, approximating the sheet direction in the LV subendocardium and subepicardium. 3rd eigenvector tracts in the inner and outer fiber strip were oriented in a radial arrangement in a short‐axis view, approximating the sheet‐normal direction in the LV myocardium that reflect the general preference of sheet orientation parallel to the myocardial wall. In contrast, the 2nd and 3rd eigenvectors in the middle strip were aligned in a radial and through‐plane orientation with respect to a short‐axis view. Supporting research data will be made available upon request.

## Discussion

The first goal in designing the phantom was to simulate values of mean ADC and FA found in myocardial tissue, and to demonstrate temporal stability in their measurement. Larger pore sizes relative to a previous study were specified to account for the higher ADC and lower FA in cardiac tissue relative to brain white matter.^12^ We report values comparable to ex vivo mouse and rat cardiac data reported in the literature, where mean ADC ranged from 0.57–0.89 × 10^‐3^ mm^2^/s, and FA ranged from 0.25–0.40,^16^, ^17^ and show the stability of these measurements over 4 months.^18^

We assessed the geometry of the fiber strips based on their nominal thickness and measured mean helix angle, and found that the effective radii of curvature of the inner, middle, and outer strips were 273 mm, 4.88 mm, and 526 mm, respectively. The tighter radius of curvature of the middle strip meant that additional force was required to overcome its inherent stiffness, to wind and secure it around the spindle. The extra force resulted in greater compression of the middle layer relative to the inner and outer layers. This is likely to have accounted for its thinner geometry, and lower b = 0 image intensity and lower mean ADC as some cyclohexane was forced out of the fibers. The compressed fibers may have taken on a more elliptical cross‐section, thereby increasing the observed FA. As the section of the fiber strip used for SEM was separate and not compressed, this ellipticity is not reflected in the SEM data. Further analysis showed that regions with higher mean ADC corresponded to regions with lower FA, and that this occurred at the edges of the strips, and could be attributed to partial volume effects. This could be mitigated by using thicker fiber strips or increasing the image resolution.

Fiber tracking in the three layers of the phantom, based on the 1st eigenvector, recreated the transmural transition in helix angle observed in the LV. Fiber tracking in the inner and outer layer, based on the 2nd and 3rd eigenvectors corresponded to the sheet and sheet‐normal orientations observed in the LV. During the manufacturing process, a charged jet of polymer solution is accelerated by electrostatic forces from a spinneret towards an oppositely charged rotating drum collector. The momentum of the polymer jet, coupled with gravity, results in slight flattening of the fibers, such that their cross‐sections are slightly elliptical, with the long and short axes of the ellipse aligned along the fiber width and depth, respectively. This imparts a sheet‐like microstructure to the fiber strip, giving rise to the observed 2nd and 3rd eigenvector orientations. The degree of flattening depends on the voltage used, the distance between the needle and the drum, the flow rate of the polymer and solution solvents, and the diameter and rotation speed of the drum.

Different behavior was observed in the middle strip, where the 2nd and 3rd eigenvectors were oriented in a radial and through‐plane manner with respect to a short‐axis slice. We hypothesize this was a result of using a filament to secure the fiber strip. As the filament was wound around the fiber strip in a helical manner, it restricted diffusion primarily across the width of the fiber strip, resulting in the 2nd and 3rd eigenvectors being oriented along the depth and width of the strip instead. This effect was greatly enhanced relative to the inner and outer layers, as the filament securing the middle layer was wound more tightly and densely to overcome the higher bending resistance.

Cardiac motion is a major challenge in in vivo cardiac diffusion MRI. This necessitates the use of methods, such as cardiac and respiratory gating, stimulated echo, and higher‐order moment‐nulling diffusion gradients, to mitigate the sensitivity of the pulse sequence to motion. The periodic deformation of the heart also introduces regional variations in strain over the cardiac cycle, distorting the phase modulation introduced by the diffusion weighting. In addition, in vivo acquisitions are limited in temporal resolution, spatial coverage, and choice of diffusion weighting scheme. Further technical developments are needed to take advantage of more sophisticated diffusion MRI methods for investigating tissue microstructure, and to enhance its prospects for clinical application. A dynamic cardiac phantom with flexible microstructure, and fully controllable and physiological deformation, would serve as an ideal test bed for development of such sequences. One study compared the performance of several diffusion weighting schemes in the presence of motion by using a rotating pig spinal cord phantom.^19^ However, more representative cardiac phantoms, both in terms of morphology and motion, have yet to be developed.

There are two main limitations in this study. 1) Lack of contractility: our experience has shown that it is feasible to modify the composition of the polymer fibers to increase the elasticity of our phantom, so that it might undergo periodic deformations on the order experienced in cardiomyocytes. Tuning the polymer composition and implementing a dynamic actuating mechanism is the subject of further work. 2) Use of three discrete layers of prefabricated fiber strips that are bent at different radii of curvature, leading to variable stresses, strains, and diffusion properties. In this respect, a continuum of fibers could be deposited and wound directly on the polymer spindle with a physiological helix angle distribution. This would avoid introducing variable stresses and strains, and would also preclude gaps between fiber strips that contribute to partial volume effects. Direct winding onto the spindle would also improve control over the ellipticity of the cross‐section of the fibers, enabling better specification of the degree of orthotropy and the sheet‐like behavior in the fiber strips.

Notwithstanding developments in in vivo cardiac diffusion MRI, there are many ex vivo applications of this technique that would benefit from the use of static phantoms for sequence testing and evaluation. Such ex vivo applications often sample k‐ or q‐space at a resolution and to an extent that is not feasible in vivo, and thus provide complementary information. Examples include high‐resolution DTI in mouse hearts.^17^ and diffusion spectrum imaging in infarcted rat hearts 20

The design of a phantom needs to balance mimicking biological structure, reproducibility in manufacture, and amenability to characterization and validation. On the one hand, it could be argued that the phantom should recapitulate the branching syncytium and discrete sheet arrangement found in the myocardium. However, such a phantom would be difficult to consistently reproduce, and hard to characterize in a meaningful way. Crucially, this degree of complexity (eg, degree of branching and spatial separation of sheets) cannot be explicitly measured with DTI. Our design approach was instead premised on 1) reproducibility in manufacture, 2) compatibility with SEM characterization, and 3) sufficient structural complexity, through the use of customizable hollow fibers, to mimic biophysical diffusion properties as measurable with DTI.

In conclusion, a biomimetic phantom simulating diffusion in the heart was designed and built by co‐electrospinning. Physiologically relevant values of mean ADC and FA were reported, and these were shown to be stable over 4 months. Fiber tracking showed that the phantom approximated the fiber and sheet orientations found in the LV. It is envisaged that the phantom could aid development and validation of new techniques for investigating cardiac architecture and microstructure based on diffusion MRI, reduce the numbers of animals and patients needed in methods development, and improve quality assurance in longitudinal and multicenter cardiac diffusion MRI studies.
